# Oxygen targets in patients with septic shock: a retrospective cohort study on the association between hyperoxia and mortality

**DOI:** 10.3389/fmed.2025.1603926

**Published:** 2025-08-25

**Authors:** Louisa T. Lalla, Anika Luise Lange, Nils Schweingruber, Tim T. Hardel, Maria Schröder, Stefan Kluge, Jörn Grensemann

**Affiliations:** ^1^Department of Intensive Care Medicine, University Medical Center Hamburg-Eppendorf, Hamburg, Germany; ^2^Department of Neurology, University Medical Center Hamburg-Eppendorf, Hamburg, Germany

**Keywords:** septic shock, sepsis, intensive care, critical ill, hyperoxia, hypoxia, oxygenation, arterial oxygen partial pressure

## Abstract

**Background:**

In critically ill patients with septic shock, adequate oxygenation is crucial and hypoxia should be avoided. However, hyperoxia has been linked to the formation of reactive oxygen species, inflammation, and vasoconstriction, which could potentially harm critically ill intensive care patients. Therefore, this study aimed to examine the association between oxygen exposure and mortality and to define optimal oxygen target ranges for this specific group of patients.

**Methods:**

This retrospective, single-center cohort study examined the influence of arterial oxygen partial pressure (PaO_2_) on in-hospital mortality in intensive care unit (ICU) patients with septic shock. Time-weighted mean PaO_2_ values for days 1, 2–3, 4–7, and 8–14 were calculated and analyzed using multivariable binary logistic regression models and relative distribution analyses, adjusting for age and sepsis-related organ failure assessment (SOFA) score on day 1. Additionally, PaO_2_ integrals above thresholds of 80, 100, 120, and 150 mmHg were calculated for periods from admission up to days 1, 3, 7, and 14, with multivariable adjusted binary logistic regression analyses performed.

**Results:**

A total of 2,647 cases from 2,463 patients, identified between January 2016 and December 2022, met the inclusion criteria. The time-weighted mean PaO_2_ values associated with the lowest mortality were 92, 81, 83, and 85 mmHg for days 1, 2–3, 4–7, and 8–14, respectively. The optimal oxygen target range decreased over time: from 77 to 103 mmHg on day 1 to 72 to 90 mmHg on days 2 and 3, and to 74 to 92 mmHg for days 4 to 7. Additionally, PaO_2_ integrals above all set thresholds of 80, 100, 120, and 150 mmHg for all periods were found to be independently associated with increased in-hospital mortality (*p* < 0.05 for day 1; *p* < 0.001 for up to days 3, 7, and 14).

**Conclusion:**

In this cohort, the PaO_2_ oxygen target range associated with the lowest mortality in patients with septic shock was approximately 80–105 mmHg on the first day of treatment, decreasing to approximately 75–90 mmHg during intensive care therapy.

## Background

In patients with sepsis and septic shock, adequate tissue oxygenation is essential to prevent further organ damage from hypoxia. Septic shock, the most severe form of sepsis, is characterized by an imbalance between oxygen supply and demand, which ultimately leads to tissue hypoxia. This can be primarily attributed to an impairment of microcirculatory function rather than a lack of oxygen in the blood (1, 2). Nevertheless, arterial oxygen partial pressure (PaO_2_) is a fundamental factor influencing oxygen delivery, along with the cardiac function and the hemoglobin level (3). An early and effective intensive care treatment, potentially requiring oxygen supplementation and mechanical ventilation, is therefore of paramount importance to reduce mortality and morbidity associated with this severe and life-threatening pathology (4).

Due to the sigmoidal binding curve between oxygen and hemoglobin, only small amounts of additional oxygen are bound to hemoglobin above a PaO_2_ of 80 mmHg (5). From this theoretical point of view, a PaO_2_ between 65 mmHg and 80 mmHg, corresponding to a peripheral oxygen saturation (SpO_2_) of approximately 91 to 96% (6), may be sufficient for adequate oxygenation in healthy subjects, while the targets in sepsis and septic shock are unknown. The current German guidelines for acute respiratory insufficiency (7) and oxygen therapy in the acute care of adult patients (3) recommend PaO_2_ ranges of 90–94% and 92–96%, respectively. With regard to oxygenation levels above those recommendations, a recent systematic review from the Cochrane Library found an increase in mortality and morbidity with hyperoxia in the overall population of intensive care patients (8). Additionally, a meta-analysis comparing liberal and conservative oxygen therapy in acutely ill patients showed an increase in mortality if oxygen is supplemented liberally (9).

Liberal oxygen supplementation may be associated with various side effects, including vasoconstriction, an inflammatory response, and an increased production of reactive oxygen species (ROS) (10). ROS are suspected to be a major contributor to oxygen toxicity through a time-and dose-dependent accumulation in long-term hyperoxia, potentially causing cell damage by apoptosis or even necrosis (11, 12). On the other hand, they may also enhance the response of the cellular immune system toward pathogens (13, 14), and therefore, an early period of short-term hyperoxia might be beneficial in ICU patients with severe infections (15–17). However, recent studies with patients suffering from sepsis have not been able to define an individual optimal PaO_2_ value or target range for this subgroup of patients (18). Similarly, sepsis guidelines do not provide any recommendations regarding arterial oxygen partial pressure or peripheral oxygen saturation (4, 19). The purpose of the present study was to investigate the relationship between PaO_2_ and clinical outcome in ICU patients with septic shock and to evaluate potential thresholds for optimal oxygen target ranges over time during intensive therapy, postulating a potential change in oxygenation targets.

## Methods

### Ethical standards

The study was performed in accordance with the ethical standards as written in the 1964 Declaration of Helsinki and its later amendments or comparable ethical standards. The retrospective and anonymized data collection and analysis were conducted in accordance with local government law (HmbKHG. §12) without the requirement for approval or informed consent.

### Study design

This was a retrospective, single-center, exploratory cohort study. The study complies with the Strengthening the Reporting of Observational Studies in Epidemiology (STROBE) reporting guidelines.

### Setting and patients

This study was conducted at the Department of Intensive Care, University Medical Center, Hamburg-Eppendorf, with a total of 140 intensive care beds on 12 wards, including the entire spectrum of adult intensive care medicine. Patients admitted to the ICU from January 2016 to December 2022 were included if the International Classification of Diseases 10th revision (ICD-10) code R57.2 for septic shock (20) was coded in the case management system, and the following criteria, according to the SEPSIS-3 definition (21), were met on the first day in intensive care: Sequential Organ Failure Assessment (SOFA) (22) score of 2 points or higher, lactate greater than 2 mmol/l, and administration of catecholamines. To ensure a valid calculation of time-weighted oxygenation parameters, patients with less than three documented arterial blood gas analyses (ABG) during intensive care were excluded from the study.

### Data retrieval

Patient cases were obtained from the central case management system (SAP, Walldorf, Germany). Data were subsequently extracted from the electronic intensive care patient data management system (Intensive Care Manager, V10, Drägerwerk, Lübeck, Germany) with its corresponding proprietary data extraction tool (ICMiq, V1.3, Drägerwerk, Lübeck, Germany). Oxygen partial pressure and time of measurement of all conducted arterial blood gas analyses, and the following demographic and descriptive data were collected: age, sex, height, weight, length of stay in the ICU, SOFA score and lactate on day 1, medical specialty, and in-hospital mortality. Data management was performed using Microsoft Excel 2019 (Microsoft Corp., Redmond, WA, United States).

### Oxygen parameters

As published previously (23), time-weighted mean PaO_2_ and PaO_2_ integrals above thresholds of 80, 100, 120, and 150 mmHg were calculated based on the ABG analysis results, assuming a linear change of PaO_2_ between two measurements (as illustrated in Supplementary Figure S1). Time-weighted mean PaO_2_ was calculated (A) for the first 24 h after admission as the hyperacute phase, (B) from 24 h post-admission up to 72 h as the acute phase, (C) from day 4 up to day 7, and (D) from day 8 to day 14 for long-term observation. To address the time-and dose-dependent accumulation of oxygen toxicity, the integrals above the set thresholds were calculated from admission to the ICU to the end of (A) 24 h, (B) 72 h, (C) day 7, and (D) day 14. To allow for comparisons, all parameters were calculated as mean values per day. If less than three ABGs were obtained in the particular period of time, no oxygen parameters were calculated. All calculations were performed with Visual Basic for Applications (V7.1, Microsoft Corp., Redmond, WA, United States).

### Outcome parameters

The primary outcome of this study was in-hospital mortality. For patients who were admitted to the ICU multiple times and died during or following their most recent stay, the outcome of the previous admissions was defined as survival.

### Statistical analysis

Univariate statistical analyses were conducted using Student’s *t*-test on patient characteristics and oxygenation parameters. Age and SOFA score on day 1 were selected *a priori* as covariates for all multivariable statistical analyses. To assess time-weighted mean PaO_2_ as a continuous parameter and to determine the non-linear correlation between oxygen exposure and in-hospital mortality, multivariable adjusted binary logistic regression models using restricted cubic splines with knots at the 10th, 50th, and 90th percentiles were generated and illustrated as odds ratios with corresponding 95% confidence intervals, as published previously (24). Additionally, relative distribution analyses (25), based on the time-weighted mean PaO_2_, were carried out to determine lower and upper thresholds with corresponding 95% CI of potential optimal oxygen target ranges. Changes in the optimal oxygen target range during the treatment were investigated using the Welsh test. For the multivariable analyses of PaO_2_ integrals above the thresholds, binary multivariable logistic regression analyses for in-hospital mortality as the dependent variable were calculated, and the results were represented as odds ratios (OR) along with the corresponding 95% confidence intervals (CI). Separate models were calculated for different oxygenation parameters as independent variables. Statistical tests were considered statistically significant at a *p*-value of <0.05. If not stated otherwise, data are given for cases of sepsis therapy rather than the number of patients. All statistical analyses were performed with R Project for Statistical Computing (version 4.3.3, R Foundation for Statistical Computing, Vienna, Austria). Data are given as numbers (percentage, %) for categorical parameters and mean (± standard deviation, SD) or median [interquartile range, IQR] for continuous variables, as appropriate.

## Results

From January 2016 to December 2022, the ICD-10 code R57.2 for septic shock was coded in 4,701 cases. Of these, 1,938 cases were excluded for not meeting the SEPSIS-3 criteria, and 116 cases were excluded due to missing ABG values. Ultimately, 2,647 cases from 2,463 patients, with 414,889 available ABGs, met the inclusion criteria and were included in the subsequent analyses. From these patients, 144 received treatment for septic shock more than once during the study period, resulting in more cases of sepsis therapy than individual patients. Of the included patients, 1,477 (60%) died during the hospital stay. A summary of patients’ baseline characteristics is provided in Table 1. The time-weighted mean PaO_2_ values were 92 ± 23, 83 ± 16, 82 ± 12, and 82 ± 12 mmHg on days 1, 2–3, 4–7, and 8–14, respectively. An illustration of the distribution of time-weighted mean PaO_2_ values is given in Supplementary Figure S2.

**Table 1:** Baseline characteristics.

Baseline characteristics	Overall (*n* = 2,647)	Survived (*n* = 1,170)	Deceased (*n* = 1,477)
Age [years]	64 ± 15	62 ± 15	65 ± 14
Sex			
Female	908 (34%)	393 (34%)	515 (35%)
Male	1739 (66%)	777 (66%)	962 (65%)
Height [cm]	174 ± 13	174 ± 10	174 ± 16
Weight [kg]	82 ± 24	81 ± 23	82 ± 24
Length of stay in intensive care unit [days]	16 ± 23	20 ± 25	13 ± 21
SOFA score on day 1	13 [5]	12 [4]	14 [5]
Lactate on day 1 [mmol/l]	6.9 ± 5.3	5.2 ± 3.5	8.4 ± 6.0
Medical specialty			
Internal medicine	1,105 (42%)	464 (40%)	641 (43%)
Surgery	1,458 (55%)	665 (57%)	793 (54%)
Neurology	50 (2%)	24 (2%)	26 (2%)
Other	34 (1%)	17 (1%)	17 (1%)
In-hospital mortality ^*^	1,477 (60%)		

Data are given as numbers and percentages, mean ± standard deviation, or median and interquartile range in brackets, as applicable. ^*^ referring to the number of patients (*n* = 2,463) instead of the quantity of intensive care episodes.

**Figure 1:**
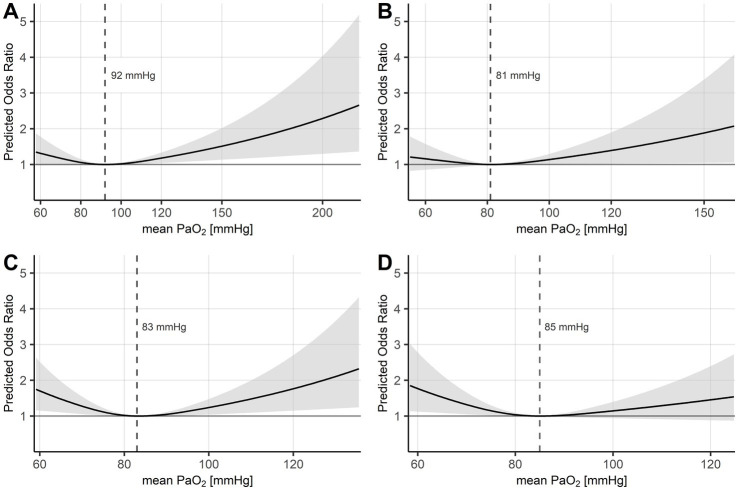
Logistic regression models of mean PaO_2_ for in-hospital mortality. Logistic regression models for in-hospital mortality modelling time-weighted mean PaO_2_ in mmHg as a restricted cubic spline with three knots at the 10th, 50th, and 90th percentiles. The gray area represents 95% confidence intervals (*p* < 0.05). Models were adjusted for age and sepsis-related organ failure assessment score on day 1. The dashed vertical line represents the reference PaO_2_ associated with the lowest mortality. **(A)** On day 1: reference: PaO_2_ = 92 mmHg, significance for hyperoxia: PaO_2_ = 106 mmHg. **(B)** Days 2 and 3: reference: PaO_2_ = 81 mmHg, significance for hyperoxia: PaO_2_ = 100 mmHg. **(C)** Days 4 to 7: reference: PaO_2_ = 83 mmHg, significance for hypoxia/hyperoxia: PaO_2_ = 77/93 mmHg. **(D)** Days 8 to 14: reference: PaO_2_ = 85 mmHg, significance for hypoxia: PaO_2_ = 77 mmHg. PaO_2_ = arterial partial pressure of oxygen in mmHg.

The univariate analysis based on the time-weighted mean PaO_2_ for the four periods of time showed no significant impact of PaO_2_ on in-hospital mortality (Supplementary Table S1). To address PaO_2_ as a continuous parameter, time-weighted mean PaO_2_ values for all four periods of time were depicted in separate multivariable adjusted logistic regression models, revealing a U-shaped association between oxygenation and in-hospital mortality with an increased occurrence of adverse outcomes for both hypoxia and hyperoxia. The PaO_2_ values associated with the lowest in-hospital mortality were 92 mmHg, 81 mmHg, 83 mmHg, and 85 mmHg for days 1, 2–3, 4–7, and 8–14, respectively (Figures 1A–D). Statistical significances (*p* < 0.05) toward hyperoxia were achieved at 106 mmHg, 100 mmHg, and 93 mmHg for days 1, 2–3, and 4–7, respectively (Figures 1A–C). In addition, hypoxia below 77 mmHg from day 4 to day 14 was statistically significantly (*p* < 0.05) associated with an increase in mortality (Figures 1C,D). Beyond these limits, a further increase in the odds ratios for in-hospital mortality could be observed with more pronounced hypoxia or hyperoxia.

**Figure 2:**
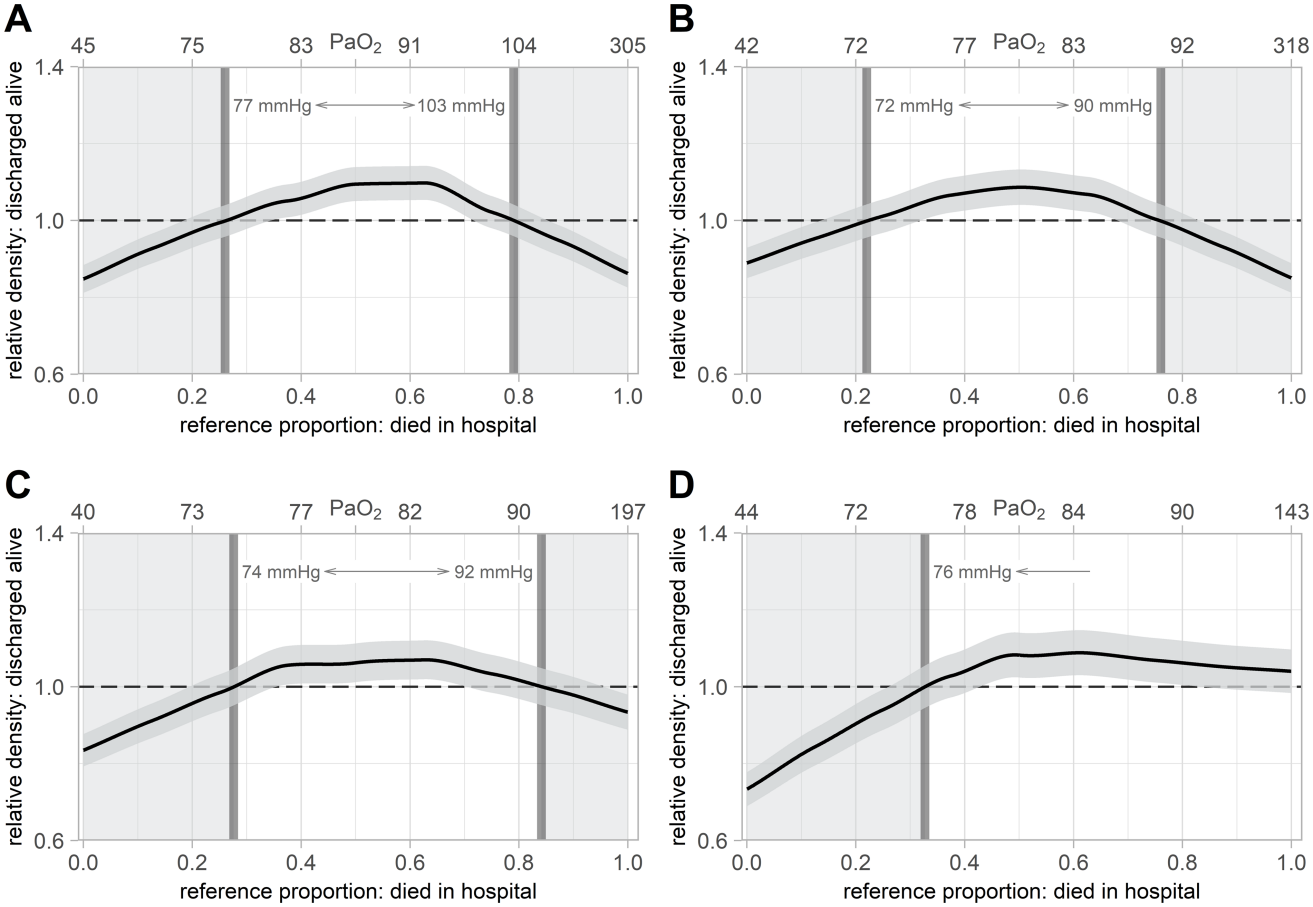
Relative distribution analyses of mean PaO_2_ for in-hospital mortality. Relative distribution analysis for in-hospital mortality, based on time-weighted mean PaO_2_, adjusted for age and sepsis-related organ failure assessment score on day 1. The upper x-axis displays the time-weighted mean PaO_2_ corresponding to the reference proportion. The gray area depicts the 95%CI. PaO_2_ = arterial partial pressure of oxygen. 95%CI = 95% confidence interval. **(A)** On day 1. PaO_2_ of lower threshold: 77 mmHg = 1.00, 95%CI = 0.959–1.041 (PaO_2_: 74–81 mmHg). PaO_2_ of upper threshold: 103 mmHg = 1.00, 95%CI = 0.959–1.041 (PaO_2_: 98–110 mmHg). **(B)** Days 2 and 3. PaO_2_ of lower threshold: 72 mmHg = 1.00, 95%CI = 0.957–1.043 (PaO_2_: 70–75 mmHg). PaO_2_ of upper threshold: 90 mmHg = 1.00, 95%CI = 0.957–1.043 (PaO_2_: 86–93 mmHg). **(C)** Days 4 to 7. PaO_2_ of lower threshold: 74 mmHg = 1.00, 95%CI = 0.952–1.048 (PaO_2_: 72–76 mmHg). PaO_2_ of upper threshold: 92 mmHg = 1.00, 95%CI = 0.952–1.048 (PaO_2_: 86–103 mmHg). **(D)** Days 8 to 14. PaO_2_ of lower threshold: 76 mmHg = 1.00, 95%CI = 0.945–1.055 (PaO_2_: 74–79 mmHg). PaO_2_ of upper threshold: no threshold evaluated.

As upper and lower thresholds for an optimal oxygen target range for intensive care therapy of patients with septic shock, the relative distribution analyses revealed 77 mmHg (95%CI: 74 mmHg to 81 mmHg) and 103 mmHg (95%CI: 98 mmHg to 110 mmHg) for day 1, 72 mmHg (95%CI: 70 mmHg to 75 mmHg) to 90 mmHg (95%CI: 86 mmHg to 93 mmHg) for days 2 and 3, and 74 mmHg (95%CI: 72 mmHg to 76 mmHg) to 92 mmHg (95%CI: 86 mmHg to 103 mmHg) for days 4 to 7 (Figures 2A–C). For long-term intensive care therapy (days 8 to 14, Figure 2D), the lower threshold was at PaO_2_ above 76 mmHg (95%CI: 74 mmHg to 79 mmHg), whereas no upper threshold could be identified by the relative distribution analysis. The upper thresholds of the optimal oxygen target ranges decreased significantly over time (days 2 to 3 vs. day 1: *p* < 0.001, days 4 to 7 vs. day 1: *p* = 0.038).

**Figure 3:**
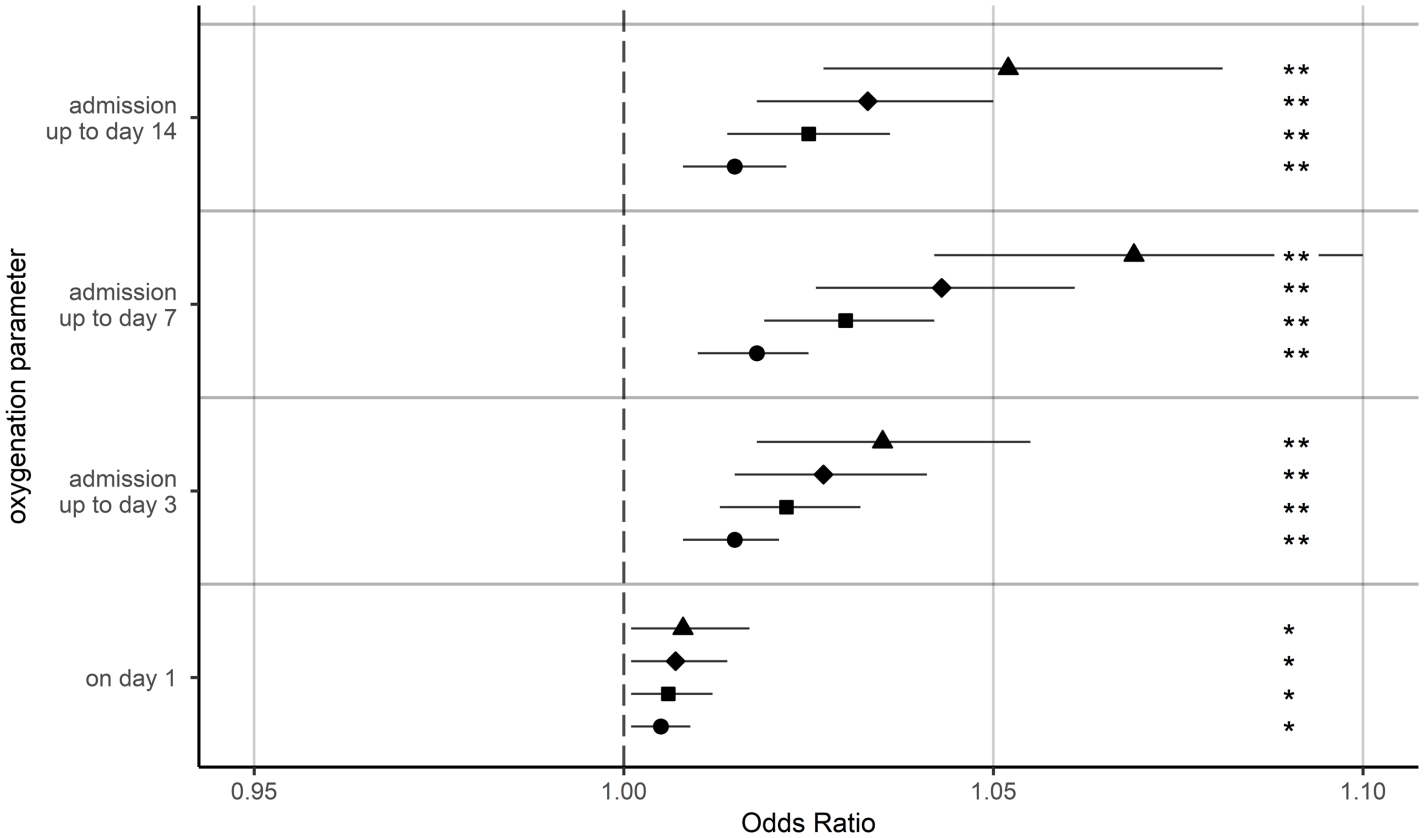
Odds ratios for in-hospital mortality. Odds ratios for in-hospital mortality for the integrals above the respective threshold, depicting a mean increase of 1 mmHg/day. Separate binary logistic regression models were calculated for each oxygenation parameter. All models were adjusted for age and sepsis-related organ failure assessment score on day 1. ▲ = arterial oxygen partial pressure integral above a threshold of 150 mmHg. ♦ = arterial oxygen partial pressure integral above a threshold of 120 mmHg. ■ = arterial oxygen partial pressure integral above a threshold of 100 mmHg. ⬤ = arterial oxygen partial pressure integral above a threshold of 80 mmHg. *p < 0.05 versus baseline. **p < 0.001 versus baseline. Error bars present the 95% confidence intervals.

With regard to the integrals above thresholds of 80, 100, 120, and 150 mmHg, hyperoxia above all thresholds could be determined as a predictive factor for mortality in univariate analyses on day 1 (*p* < 0.05) as well as up to days 3, 7, and 14 (*p* < 0.001) (Supplementary Table S2). After adjusting for age and SOFA score on day 1, these results could be confirmed in multivariable binary logistic regression analyses, showing significantly higher odds ratios for mortality with higher PaO_2_ values (Figure 3, Supplementary Table S3).

## Discussion

In this retrospective study of patients suffering from septic shock, both hypoxia and hyperoxia were associated with an increase in in-hospital mortality in this specific subgroup of ICU patients. The relationship between oxygen exposure and outcome exhibited a U-shaped curve with an optimal arterial oxygen partial pressure occurring at an intermediate range. Interestingly, the target range associated with the best outcome decreased after the first 24 h following the onset of shock. Using integral calculations above multiple thresholds to assess the relationship between oxygen exposure and oxygen toxicity, hyperoxia exceeding 80 mmHg was associated with an increase in in-hospital mortality from admission up to day 14. Of note, oxygen integrals above the lower thresholds also include the integrals above the higher thresholds; therefore, the optimal PaO_2_ revealed by the logistic regression models or the optimal target range identified in the relative distribution analyses may be within or above the lower integral thresholds (24). Our results favor a slightly higher PaO_2_ than that currently recommended in guidelines, which advise a target range for oxygen saturation of 92–96% in the acute care of adult patients (3), corresponding to a PaO_2_ of 68 mmHg to 84 mmHg (6), and of 90–94% in the mechanically ventilated patients (7), corresponding to a PaO_2_ of 60 mmHg to 76 mmHg (6).

The main goal of oxygen administration in intensive care therapy is the prevention of hypoxic organ damage by ensuring sufficient tissue oxygenation while minimizing oxygen toxicity (26). As outlined above, only negligible amounts of oxygen are bound to hemoglobin above a PaO_2_ of 80 mmHg, corresponding to an SpO_2_ of approximately 96% (6). However, supranormal PaO_2_ may have other effects beyond cell oxygenation (26), which may be beneficial or harmful to the ICU patient, depending on the underlying pathology (10); for sepsis, it has been shown that higher oxygenation targets on the first days might be beneficial (15–17, 27). One reason may be that neutrophils rely on reactive oxygen species (ROS) for their bactericidal effects and are therefore capable of producing and releasing ROS to directly damage the pathogens by impairing, inter alia, their genetic material, proteins, and cell membranes (13, 14). Increasing the oxygen partial pressure may facilitate this mechanism, especially on the first day of treatment.

On the other hand, hyperoxia induces a number of physiological disturbances, such as oxidative stress, inflammation, and vasoconstriction, which collectively result in oxygen toxicity (26). While oxygen radicals are produced permanently, an excess may overwhelm the antioxidative mechanisms of the body and therefore lead to toxic effects (11), e.g., damage to cell membranes, proteins, DNA, and mitochondria. Ultimately, this oxidative stress may promote cell death through apoptosis or necrosis, leading to systemic tissue and organ damage (28). In addition to oxidative stress, hyperoxia may lead to a further inflammatory reaction through an activation of immune cells and the release of pro-inflammatory cytokines, e.g., interleukin 6 (29), which may contribute to neuroinflammation (30), pulmonary damage (30–32), and vasoconstriction (33), causing a secondary deterioration of tissue oxygenation (34).

After day 1, the oxygen target range associated with the lowest mortality decreased to approximately a PaO_2_ of 80 mmHg. We suggest that, with concurrent appropriate antibiotic therapy, less ROS are required to facilitate neutrophil killing and that the balance of tissue oxygenation and oxygen toxicity reaches a lower tipping point than on day 1. For long-term hyperoxia during days 8 to 14 of intensive care therapy, we could not evaluate an upper threshold for an optimal oxygen target range in ICU patients with septic shock, presumably due to fewer cases with available ABGs until day 14 due to deceased or discharged patients. Nevertheless, a U-shaped association between PaO2 and mortality persisted, with higher PaO_2_ tending to be associated with a worsened outcome.

To date, several prospective studies have investigated the influence of hyperoxia on the mortality of ICU patients with sepsis or septic shock and attempted to define an optimal oxygen target range for this specific patient population. Although the HYPERS2S trial did not show a significant difference in mortality for patients ventilated with a fraction of inspired oxygen (FiO_2_) of 1.0 and those targeted to an SpO_2_ of 88–95% for the first 24 h of treatment, it was prematurely terminated due to complications in the hyperoxia group (35). A *post-hoc* analysis of this trial found that hyperoxia was associated with an increase in mortality in patients with a lactate concentration greater than 2 mmol/l (36), aligning with the result of our study. These findings are consistent with those of other studies, which have demonstrated an increase in mortality if the PaO_2_ at ICU admission was above 150 mmHg in patients with pre-hospital invasive ventilation (37) or greater than 300 mmHg in the initial treatment of severe infections (38). In contrast, some observational studies were able to show beneficial effects of hyperoxia in septic ICU patients with a decrease in mortality when the PaO_2_ was above 100 mmHg (15, 17) or greater than 80 mmHg (16). One study could depict a U-shaped relationship between PaO_2_ and the probability of death, with the optimal PaO_2_ at 300 mmHg (39), which is considerably higher than the optimal PaO_2_ indicated by our data. Some retrospective studies have been unable to demonstrate a statistically significant influence of hyperoxia on the mortality in sepsis patients (40–44). To date, only one randomized trial has examined three oxygen target ranges simultaneously to test for a U-shaped relationship between oxygen dose and outcome, but this single-center study included ventilated medical patients, only approximately 30% of whom had sepsis or septic shock, and failed to show a difference between groups (45). According to a recent systematic review, the optimum ranges of oxygen levels in sepsis and septic shock remain unknown, concluding the need for further research on this topic (18). Similarly, our findings emphasize the necessity for further studies, especially randomized controlled trials and fundamental research.

The mortality rate in our cohort was above the previously published rate for patients suffering from septic shock (46). We attribute this high rate to our function as a tertiary care center, providing specialized care to critically ill patients with particularly complex health conditions.

This study has certain limitations. The retrospective design enabled us to demonstrate an association between oxygen exposure and in-hospital mortality in patients with septic shock; however, this does not imply causality. Potential confounders biasing the results or underlying reasons for hyperoxygenation cannot be ruled out. The calculation of mean values from numerous ABGs during the ICU treatment allowed us to explore the effect of long-term oxygen exposure, but the occurrence of temporary hypoxia, for example, during acute pulmonary complications, or short-term hyperoxia, such as during preoxygenation before interventions, between the measurements cannot be excluded. Furthermore, with an extended period of ABG-guided ventilation, the number of patients exhibiting supranormal arterial oxygen partial pressure values decreased because normoxia was targeted in accordance with the preexisting literature on hyperoxia in ICU patients (47).

## Conclusion

In this retrospective cohort study exploring the association between oxygen exposure and mortality in ICU patients with septic shock, the relationship between PaO_2_ and in-hospital mortality appeared U-shaped, with adverse effects of hypoxia as well as hyperoxia. Notably, oxygen ranges associated with the lowest mortality decreased from approximately 80 to 105 mmHg on day 1 to approximately 75 to 90 mmHg in the further course of treatment. These findings support the necessity of targeted oxygen supplementation, considering oxygen as a vital medication with the potential for dose-and time-dependent adverse effects. Further prospective clinical trials should take into account changing target ranges during the treatment of ICU patients with septic shock to improve patient outcomes.

## Data Availability

The raw data supporting the conclusions of this article will be made available by the authors, without undue reservation.
